# Navigating End-of-Life Decisions With Amyotrophic Lateral Sclerosis: A Patient-Centered Perspective on the Clinical and Legal Barriers to Medical Aid in Dying

**DOI:** 10.7759/cureus.92254

**Published:** 2025-09-13

**Authors:** Diane Marie, Larry E Miller, Samir K Bhattacharyya, Frederick M Frankhauser

**Affiliations:** 1 Research, Independent Research, Santa Ana, USA; 2 Clinical Research, Miller Scientific, Johnson City, USA; 3 Research, Independent Research, Orlando, USA; 4 Pharmaceutical Business and Administrative Sciences, Massachusetts College of Pharmacy and Health Sciences, Boston, USA

**Keywords:** als, amyotrophic lateral sclerosis, disability, maid, medical aid in dying

## Abstract

Amyotrophic lateral sclerosis (ALS) is a progressive and incurable neurodegenerative disease that often leads to loss of speech, swallowing restrictions, respiratory failure, paralysis, and total physical dependence, despite preserved cognitive function. For some affected individuals, the anticipated decline in autonomy associated with ALS leads to the consideration of medical aid in dying (MAiD). In the United States (US), MAiD is legally permitted in 12 jurisdictions (11 states and Washington, DC); however, most require patients to self-administer the prescribed medications. This stipulation creates an inequitable barrier for people with late-stage ALS, as the ability to swallow medication or manipulate delivery devices is often lost well before the end of life. Patients with ALS who are considering MAiD and otherwise meet all eligibility criteria must weigh the decision to end their lives earlier than desired against the risk of prolonging life and becoming physically unable to self-administer MAiD. This paper, coauthored by a patient with bulbar-onset ALS, integrates clinical literature, legal analysis, and lived experience to examine how existing MAiD statutes in the US fail to accommodate the physical disabilities of otherwise eligible patients from using this option. Overall, legislative reform in the US is urgently needed to ensure that MAiD eligibility is based on informed consent and clinical criteria without unfair exclusion based on physical disability.

## Editorial

Patient-authored foreword

I (DM) am a person living with bulbar-onset amyotrophic lateral sclerosis (ALS). In my case, the symptoms of my disease first appeared as slurred speech and difficulty swallowing. ALS is often diagnosed by exclusion, which means that missed or delayed diagnoses are unfortunately common; this was true for me as well. I sought an explanation for my symptoms from the onset, but it took approximately nine months to receive this diagnosis. My initial evaluations with a neurologist were limited to blood work, MRI, and telehealth visits and did not lead to further testing or an explanation for my symptoms. Ultimately, at the strong recommendation of my primary care physician, I sought a second opinion from another neurologist, which led to my diagnosis in the spring of 2025, just three months ago at the time of this writing. Since then, my physical condition has deteriorated considerably. My speech is now unintelligible, making it difficult to communicate with others in my assisted living facility, and it is deeply isolating. My limited swallowing ability restricts me to pureed foods, my mobility is compromised, and I require nighttime ventilatory support (soon to be full-time). I have been told that my life expectancy is approximately nine months.

My doctors have explained that during the final six months of my life, I will be bedbound, completely dependent on others, and paralyzed inside my body, losing the ability to move anything except my eyes. Like many people with ALS, my cognitive function remains intact even as my physical abilities decline. Because of this prognosis, I have been forced to confront end-of-life decisions far earlier than I would have otherwise wished.

I reside in California, a US state where medical aid in dying (MAiD) is legal. I remain conflicted about whether I would choose MAiD, but I want the option to consider it if my suffering becomes intolerable. I currently meet all legal criteria for MAiD except one. I am an adult with an incurable and irreversible terminal illness, mentally competent and fully capable of making healthcare decisions for myself, and physically able to self-administer life-ending medications. However, under the current law, I do not qualify because no state allows MAiD unless medical professionals determine that the patient has a life expectancy of six months or less. Although this legal requirement is intended as a safeguard to ensure careful decision-making, it can also create a significant obstacle for patients who wish to consider MAiD.

As my ALS progresses, I am rapidly losing the ability to swallow and use my hands, which are the very functions required to self-administer life-ending medication. This creates a devastating dilemma because I will not qualify for MAiD until it is determined that I have less than six months to live. However, by the time I qualify, I may no longer be physically able to self-administer MAiD, effectively removing this option altogether.

This paper was born out of my lived experience with ALS and the difficult decisions it has forced me to face. We describe how current MAiD laws fail to accommodate patients like me and argue for changes that reflect the clinical and ethical realities of living, and dying, with ALS.

Background

Amyotrophic lateral sclerosis (ALS) is a progressive, incurable, and ultimately fatal neurodegenerative disease. ALS is characterized by degeneration of motor neurons in the brain and spinal cord and is often definitively diagnosed only after considerable delay [1]. Despite preserved cognitive function in most cases [2,3], ALS leads to progressive weakness of voluntary skeletal muscles, dysarthria, dysphagia, and respiratory insufficiency, with many patients completely physically dependent on others during the end stages of disease. The annual incidence of ALS ranges from 4-8 per 100,000 individuals in the US [4,5] and is anticipated to increase in the coming years [6]. The typical age at diagnosis is between 55 and 65 years [7], and the life expectancy following diagnosis is approximately two years [8], though this varies based on phenotype, age, and medical history. Bulbar-onset ALS, which begins with impairments in speech and swallowing, accounts for approximately one-third of cases and is associated with more rapid progression and shorter survival than limb-onset variants [9]. Regardless of expected survival, most patients experience loss of autonomy and function early in the disease course and spend the majority of their remaining time in a severely debilitated and often fully dependent state. In fact, when patients and caregivers rate their physical function and physical role on the EQ-5D questionnaire (0-100 scale), the median scores on both are 0 [10].

For some individuals with ALS, the anticipated loss of function and autonomy leads to consideration of MAiD. MAiD typically involves a combination of cardiotonic, opioid, and sedative medications that cause loss of consciousness (typically within five minutes) and death through respiratory depression and cardiac arrest (typically within 30 minutes) [11]. In jurisdictions where MAiD is legally available, such laws are intended to support patient autonomy and alleviate suffering. However, for patients with ALS, the clinical reality of progressive paralysis combined with legal requirements for self-administration of lethal medication creates a cruel dilemma. Approximately one-third of affected individuals express interest in MAiD if their suffering becomes intolerable [12], yet current laws require that it be initiated while patients retain the physical ability to self-administer the prescribed medication. However, patients are not legally eligible to access MAiD until it is determined they have a life expectancy of six months or less. This creates a narrow and clinically unrealistic window where waiting until they meet legal eligibility may mean they are no longer physically capable of completing the required act, regardless of prognosis, suffering, or intent. This paper examines how existing MAiD laws conflict with the clinical realities of ALS by drawing on clinical research, legal statutes, and the lived experience of a patient author.

Disproportionate impact of the MAiD self-administration requirement on ALS patients

**Figure 1:**
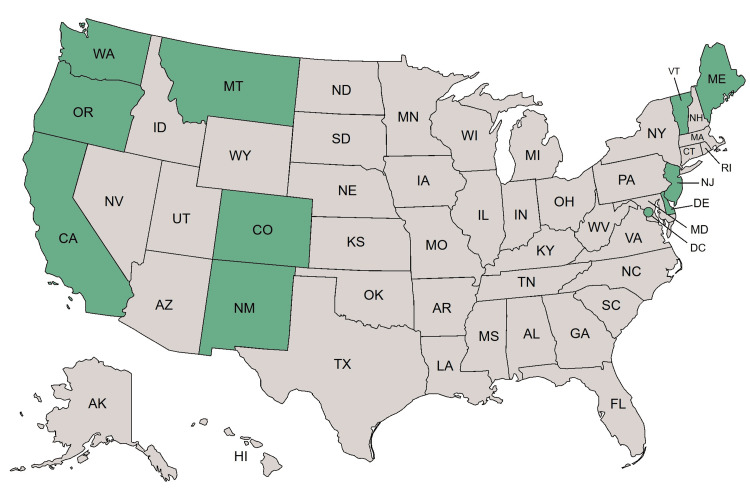
United States jurisdictions where Medical Aid in Dying (MAiD) is legally authorized as of August 2025 Green shading indicates the 12 US jurisdictions where MAiD is legally authorized. Red shading indicates jurisdictions where MAiD is not authorized. Although Delaware signed MAiD into law on May 20, 2025, it cannot be utilized until the regulations are completed or January 1, 2026, whichever comes first (original figure created by authors).

Despite growing public support [13], MAiD is legally authorized in only 12 US jurisdictions as of August 2025 (Figure 1). These laws permit terminally ill, mentally competent adults to request and self-administer physician-prescribed lethal medications. However, all include a non-negotiable requirement that the patient must perform the final act of administration independently. Self-administration is strictly interpreted as an “affirmative, conscious, and physical” action taken by the patient, without direct assistance from another individual, even if the patient is physically incapable of performing that act due to disease progression. For patients with ALS, especially those with advanced-stage disease, this requirement functions as a barrier to access.

Unlike patients with terminal cancer, who comprise the majority of MAiD users [14] and often maintain functional independence late into their disease course, individuals with ALS frequently lose the ability to swallow, use their hands, or operate assistive devices months before death. This progressive motor decline directly interferes with every legally permitted method of self-administration, including oral ingestion, syringe delivery via feeding tube, or rectal catheter insertion. As a result, patients with ALS face unique barriers to MAiD access. Despite meeting every other eligibility criterion, many will become physically incapable of carrying out the act required to receive care. Yet paradoxically, they represent a disproportionately high percentage of those who pursue MAiD. For example, in California, ALS accounts for only 0.2% of all resident deaths but 6.5% of those who accessed MAiD [15]. This greater than 30-fold overrepresentation highlights both the severity of suffering associated with ALS and the unrealistic timing decisions forced by current MAiD requirements.

The requirement for self-administration creates what is termed a “perverse incentive” structure. Patients with ALS who wish to access MAiD must closely monitor their physical decline and precisely time their request early enough to retain the ability to self-administer but late enough to meet the six-month prognosis requirement. As a result, patients may feel forced to end their lives earlier than they otherwise would, or risk waiting too long and losing the option entirely. The law, as written, does not account for progressive physical disability and effectively denies access to MAiD for patients who meet all clinical and legal criteria, based only on their loss of motor function. This exclusion raises serious concerns under disability rights law. The Americans with Disabilities Act (ADA) requires reasonable modifications to provide equal access to healthcare services. Yet patients with ALS may be denied a legal medical procedure solely because they cannot perform a physical act required by statute. This exclusion is incompatible with disability rights laws that mandate assistance to provide equal access to healthcare, where patients with ALS may be unable to access a medical procedure legally provided to more able-bodied terminally ill patients. Overall, the disconnect between legal eligibility and physical ability reflects a failure to accommodate disability in end-of-life care.

Limitations of recent MAiD policy reforms

While several states have updated their MAiD statutes in recent years, these reforms have largely focused on procedural logistics rather than addressing the fundamental barriers faced by patients with physical disabilities. In 2021, New Mexico enacted the Elizabeth Whitefield End-of-Life Options Act, which is sometimes cited as a more inclusive model because it allows advanced practice registered nurses and physician assistants to prescribe MAiD medication [16]. However, the statutory requirement that the patient must self-administer life-ending medication remains unchanged. Therefore, individuals with ALS and other neuromuscular diseases who cannot self-administer remain effectively excluded. Hawaii’s Our Care, Our Choice Act was amended in June 2023 to expand provider roles to APRNs and reduce the waiting period from 20 to 5 days (or 48 hours when survival is unlikely) [17]. While these changes improve procedural accessibility, the law does not alter the self-administration requirement. In May 2023, Vermont became the first state to eliminate its residency requirement, allowing out-of-state patients to access MAiD [18]. Although this removes one access barrier, which is especially relevant for people living near state borders, it still leaves the physical self-administration mandate intact. Across all jurisdictions, no current legislation directly addresses the barrier of requiring patients to physically administer the medication themselves. Though some reforms reduce procedural or residency hurdles, these changes do not help individuals who are mentally capable but physically unable to self-administer, which is the very population examined in this paper.

International models that allow clinician participation in MAiD

As of 2025, nine countries and several Australian states have legalized clinician-administered MAiD or euthanasia, which demonstrates that assisted death can be implemented safely and ethically without excluding patients who are physically unable to self-administer life-ending medication. The Netherlands and Belgium legalized euthanasia in 2002 by requiring a voluntary patient request, confirmation of unbearable suffering, and independent review by multiple physicians [19,20]. Luxembourg enacted nearly identical legislation in 2009 [20]. In Colombia, euthanasia was authorized in 2015 by permitting direct clinician involvement [21]. Canada legalized MAiD in 2016 and expanded access in 2021, where both self-administered and clinician-administered options are legal, though over 99% of cases are carried out by clinicians [22]. New Zealand implemented a national assisted dying law in 2021 that includes provisions for clinician administration [23]. Since 2017, six Australian states have adopted MAiD laws, most of which allow physician administration when patients cannot perform the act themselves [24]. Spain legalized euthanasia in 2021 and permits clinician-administered procedures under medical review [25]. In July 2025, Slovenia became the most recent country to pass assisted dying legislation by adopting an approach similar to other European nations that do not require self-administration [26]. Collectively, these international policies demonstrate that excluding patients based on physical ability is a policy choice, not a medical or ethical necessity.

Ethical considerations in MAiD access

International examples demonstrate that assisted dying can be practiced safely and ethically without requiring patients to retain physical autonomy. In the US, however, ethical concerns regarding MAiD remain common. Some individuals with ALS decline MAiD due to religious beliefs, cultural values, or a desire to pursue life-prolonging interventions. Others, including those in a locked-in state, report an acceptable or even high quality of life despite profound physical limitations [27]. These perspectives reflect the deeply personal nature of end-of-life decision-making and highlight that MAiD should remain an option, not a directive.

For clinicians, ethical objections to MAiD are equally strong. Many physicians decline to participate in MAiD, even when legally authorized, based on conscience, faith, or belief that it is incompatible with their role as a healer. Even among those willing to participate in principle, some are deterred by legal uncertainty, institutional policy, or professional risk [28]. Although MAiD statutes now include strict eligibility and procedural safeguards, historical associations with figures like Dr. Jack Kevorkian, who was convicted in 1999 for assisting the death of a patient with ALS, continue to influence public and professional perceptions, even though his actions would not meet the standards of current law [29].

The American Medical Association (AMA) formally opposed physician involvement in MAiD until 2019. However, their updated position now recognizes that physicians may participate according to their conscience without violating professional obligations [30]. This shift acknowledges that moral diversity exists within medicine and affirms that participation in MAiD is ethically permissible within legal bounds. Ultimately, ethical debates about MAiD will continue, but laws that exclude willing, competent patients on the basis of physical disability raise distinct concerns. Ensuring access for patients with ALS does not require abandoning ethical safeguards, but rather applying them equitably.

Conclusion

The requirement for self-administration in US MAiD statutes creates a predictable and preventable barrier for patients with ALS. While some jurisdictions have improved procedural access, most laws remain misaligned with the realities of progressive neuromuscular disease. International legislative reforms demonstrate that it is possible to uphold safeguards without excluding patients based on physical ability. Addressing this issue is not about expanding MAiD access broadly, but about eliminating a narrow structural exclusion that uniquely burdens patients who meet every other legal and ethical criterion. Individuals with ALS should not be denied a legal medical option simply because they cannot swallow a pill or press a syringe. Overall, aligning MAiD statutes with clinical realities and disability rights is not only feasible, but ethically imperative.
